# Probabilistic approaches to alignment with tandem repeats

**DOI:** 10.1186/1748-7188-9-3

**Published:** 2014-03-01

**Authors:** Michal Nánási, Tomáš Vinař, Broňa Brejová

**Affiliations:** 1 Department of Computer Science, Faculty of Mathematics, Physics, and Informatics, Comenius University, Mlynská dolina, 842 48 Bratislava, Slovakia; 2 Department of Applied Informatics, Faculty of Mathematics, Physics, and Informatics, Comenius University, Mlynská dolina, 842 48 Bratislava, Slovakia

**Keywords:** Sequence alignment, Hidden Markov model, Tandem repeat

## Abstract

**Background:**

Short tandem repeats are ubiquitous in genomic sequences and due to their complex evolutionary history pose a challenge for sequence alignment tools.

**Results:**

To better account for the presence of tandem repeats in pairwise sequence alignments, we propose a simple tractable pair hidden Markov model that explicitly models their presence. Using the framework of gain functions, we design several optimization criteria for decoding this model and describe resulting decoding algorithms, ranging from the traditional Viterbi and posterior decoding to block-based decoding algorithms tailored to our model. We compare the accuracy of individual decoding algorithms on simulated and real data and find that our approach is superior to the classical three-state pair HMM.

**Conclusions:**

Our study illustrates versatility of pair hidden Markov models coupled with appropriate decoding criteria as a modeling tool for capturing complex sequence features.

## Background

In this paper, we explore the use of pair hidden Markov models (pair HMMs, PHMMs) in improving the quality of pairwise sequence alignment in the presence of tandem repeats. We propose a simple tractable model that explicitly accounts for short tandem repeats, and we use the framework of maximum expected gain to explore a variety of decoding optimization criteria for our model.

Pair HMMs have for a long time played a major role in sequence alignment [1]. The traditional Needleman-Wunsch algorithm [2] and its variants can be easily formulated as a special case of alignment with PHMMs (we call this approach Viterbi decoding). The main advantage of PHMMs is that they allow us to express the scoring scheme in a principled way in the context of a probabilistic model.

Sequence alignments are a mainstay of comparative genomics. By comparing sequences that evolved from a common ancestor, we can infer their phylogenetic relationships, discover sites under functional constraint, or even shed light on the function of individual sequence elements. However, comparative genomic methods are very sensitive to the quality of underlying alignments, and even slight inaccuracies may lead to artifacts in the results of comparative methods.

It is very difficult to evaluate alignment accuracy, yet even simple statistics can reveal artifacts of present-day algorithms. Lunter et al. [3] demonstrated systematic biases caused by the optimization criteria set by the Needleman-Wunsch approach. They show that by using variants of the posterior decoding instead of the traditional Viterbi algorithm, one can significantly increase the alignment quality. The advantage of the posterior decoding is that it summarizes information from all alignments of the two sequences, while the Viterbi decoding seeks only one highest scoring alignment. The posterior decoding was also found superior by other authors [4-6].

An algorithm by Hudek [7] is an intermediate between the Viterbi and posterior decoding, summarizing probabilities of alignments within short blocks. The goal is to segment the alignment into blocks, where each block has gaps in only one of the two sequences. The decoding algorithm considers each block as a unit, summing probabilities of all alignments that have the same block structure. Finally, Satija et al. [8] have demonstrated that fixing a particular alignment is not necessary in some comparative genomics applications, instead one can consider all possible alignments weighted by their probability in the PHMM.

In this paper, we concentrate on modeling sequence alignments in the presence of tandem repeats. Short tandem repeats cover more than 2% of the human genome, and occur in many genes and regulatory regions [9]; in fact, majority of recent short insertions in human are due to tandem duplication [10]. Evolution of tandem repeats is dominated by tandem segmental duplications resulting in regions composed of a highly variable number of almost exact copies of a short segment. Such sequences are difficult to align with standard scoring schemes, because it is not clear which copies from the two organisms are orthologous. Misalignments due to the presence of short tandem repeats are usually not limited to the repetitive sequence itself, but may spread into neighboring areas and impact the overall alignment quality (see Section Experiments).

Sequence alignment with tandem duplication was first studied by Benson [11]. They propose an extension of the traditional Needleman-Wunsch algorithm that can accommodate tandem repeats in *O*(*n*^4^) time. They also propose several faster heuristic algorithms. Additional work in this area concentrated on computing variants of edit distance either on whole sequences with tandem arrays or on two tandem arrays using different sets of evolutionary operations [12-14].

The first probabilistic approach to alignment of tandem duplications was introduced by Hickey and Blanchette [15], who developed a new probabilistic model by combining PHMMs with tree adjoining grammars. Their model favors tandem duplications over other insertions, but the approach does not explicitly model whole arrays of tandemly repeated motifs. Moreover, algorithms to train and decode such models are relatively complex.

Some protein families (such as zinc finger proteins) contain repetitive motifs similar in nature to tandem repeats in DNA. To align such proteins, Kováč et al. [16] combined profile HMMs (capturing the properties of the repeating motif) and PHMMs (modeling alignments) into a single scoring scheme that can be decoded by a newly proposed algorithm. However, their scoring scheme is no longer a probabilistic model and the method is focused on correctly aligning individual occurrences of a single motif rather than alignment of long sequences interspersed with multiple motifs.

Here, we propose a simple tractable PHMM for sequence alignment with tandem repeats, and we explore various decoding methods for its use in sequence alignment. In addition to the classical Viterbi decoding, we define several variants of the posterior decoding as well as block-based methods tailored to the specifics of our model. To demonstrate the differences, we have implemented several of these methods and compared their performance.

## Pair HMMs for alignment with tandem repeats

Tandem repeats may arise by a complicated sequence of evolutionary events, including multiple rounds of tandem duplication, deletion, point mutation, gene conversion and other phenomena. Tandem repeat arrays at orthologous locations in two related species may have arisen in the common ancestor and thus share part of their evolutionary history, but they could be further modified by independent events occurring after speciation. Models attempting to capture such diverse evolutionary mechanisms usually lead to complex problems in inference and parameter estimation. We propose two tractable models, based on classical PHMMs, which still capture the essence of a tandem repeat array: periodically repeating motif, which may be shared between the two species, or be specific to one species only.

**Figure 1:**
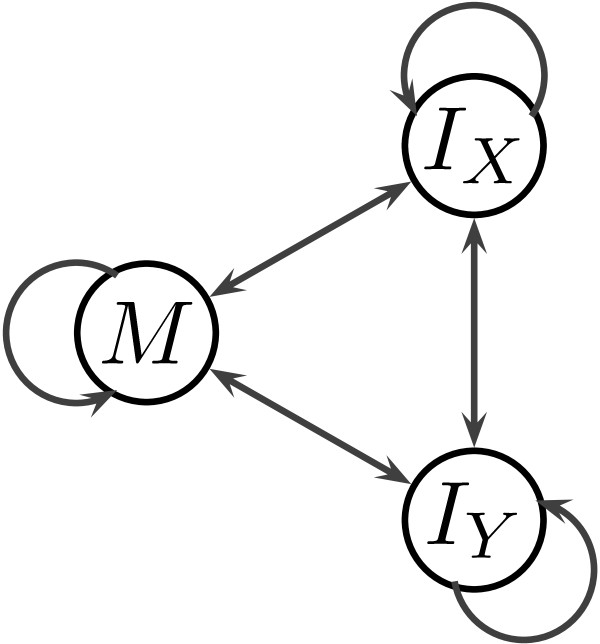
**The standard three-state PHMMs.** The match state *M* generates ungapped columns, insert states *I*_*X*_ and *I*_*Y*_ generate symbols aligned to a gap.

A PHMM defines a probability distribution over alignments of two sequences *X* and *Y*. The standard PHMM has three states (see Figure 1): match state *M* generating ungapped columns of the alignment, and two insert states *I*_*X*_ and *I*_*Y*_, where *I*_*X*_ generates alignment columns with a symbol from *X* aligned to a gap, and *I*_*Y*_ generates columns with a symbol from *Y* aligned with a gap [1]. In our work, we will use a more complex PHMM, but standard algorithms for inference are still applicable.

**Figure 2:**
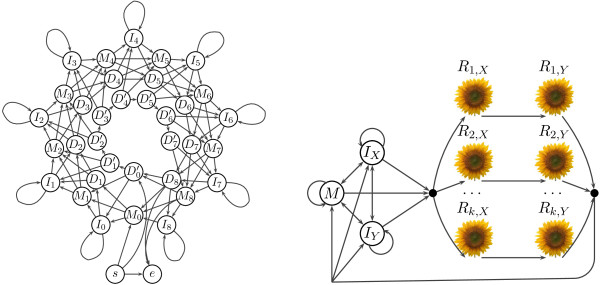
**SFF (sunflower field) model.** A pair hidden Markov model for alignment with tandem repeats. Each submodel *R*_*i*,*α*_ (left) is a circular profile hidden Markov model emitting tandem copies of the motif in one sequence. State *M*_*j*_ is the match state generating *j*th symbol of the motif, state *I*_*j*_ allows insertions between symbols *j* and *j* + 1 of the motif, and states *D*_*j*_ and *Dj*′ allow to skip state *M*_*j*_. States *s* and *e* designate the entry and exit points from the submodel. The full SFF model (right) contains a standard three-state PHMM with states *M*, *I*_*X*_ and *I*_*Y*_, and two submodels *R*_*i*,*X*_, *R*_*i*,*Y*_ for each motif *i*. States and submodels with subscript *X* and *Y* generate symbols in the respective sequence *X* or *Y* only.

We call our main model SFF, and its details are shown in Figure 2. The model contains a standard three-state PHMM and two “sunflower” submodels *R*_*i*,*X*_ and *R*_*i*,*Y*_ for each possible repeating motif *i*. Submodel *R*_*i*,*X*_ generates several (possibly zero) copies of the motif in sequence *X* and submodel *R*_*i*,*Y*_ generates motif copies in sequence *Y*. Each copy of the motif is generated independently and the number of copies in *X* and *Y* are independent and geometrically distributed.

Each sunflower submodel is a circularized profile HMM emitting copies of the motif in one of the two sequences. For a motif of length *p*, the submodel contains *p* match states *M*_0_, …, *M*_*p*-1_, each match state emitting one symbol of the motif. Insertion state *I*_*j*_ allows to insert additional characters between symbols emitted by *M*_*j*_ and *M*_(*j* + 1) mod *p*_. Deletion states *D*_*j*_ and *Dj*′ allow to bypass match state *M*_*j*_, and thus correspond to deletions with respect to the reference motif sequence. Since the submodel can emit multiple tandem copies of the motif, the states in column *p* - 1 are connected to the states in column 0. To avoid cycles consisting solely of silent states, we use two separate chains of deletion states. Chain *D*0′, …, *Dp* - 2′ can be entered only in state *D*0′, and the model can stay in this chain for at most *p* - 1 steps. Chain *D*_1_, …, *D*_*p*-1_ can be entered only after visiting at least one match or insert state in the current copy of the motif. As a result, the model can never pass around the whole circle using delete states. Note that the model prefers integer number of repeats, even though partial repeat occurrences are common in the real data. If desired, this can be addressed by simple changes in the model topology or parameters.

The overall model can have sunflower submodels for an arbitrary number of motifs; we can even define an infinite model, in which every possible finite string serves as the motif for one pair of sunflowers. In our work, we use *k*=310,091 motifs chosen as consensus strings of all tandem repeats found by the TRF program [17] run on the human chromosome 15 and its orthologous sequences in the dog genome. The probability of choosing a particular motif out of all *k* possibilities can be uniform or dependent on the motif length or composition. We assign this probability based on the observed frequency of the corresponding consensus pattern in the TRF output.

Likewise, we could use a multiple alignment of real motif occurrences to set individual parameters of the profile HMM. Instead, we use the same set of parameters for all states of all motif submodels. In particular, we set the insert and delete rates to 0.005; the match states allow mutations away from consensus according to the Jukes-Cantor model with parameter *t* = 0.05. Parameters of the three-state PHMM were estimated from the UCSC alignment of the human chromosome 15 and its orthologous regions in the dog genome.

Our model also assumes that individual copies of a fixed motif are independent. If they share part of their evolutionary history, this assumption is not valid, but it greatly simplifies the model. We could add some limited dependence by introducing repeat submodels emitting copies in the two sequences simultaneously; we have used such a model in a different setting in our previous work [16].

**Figure 3:**
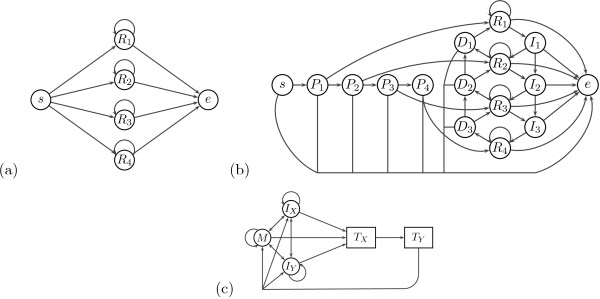
**TANTAN-like repeat model for repeat motifs of length up to *K *= 4. (a)** Model by Frith [18] representing one tandem repeat without indels. States *s* and *e* are silent, state *R*_*k*_ has emission table in which symbol at position *i* depends on symbol at position *i* - *k*. **(b)** Full model by Frith [18] allows insertions using states *I*_1_, … *I*_*k*-1_ and deletions using silent states *D*_1_, …, *D*_*K*_. Our modification includes states *P*_1_ … *P*_*K*_ for the first occurrence of the motif. **(c)** Full pair HMM consists of two copies *T*_*X*_ and *T*_*Y*_ of the model from part **(b)**, one for each input sequence, and the traditional three-state pair HMM for non-repeat regions.

We have also explored a smaller model of tandem repeats based on an approach developed for repeat masking by Frith [18]. This approach, called TANTAN, represents all repeats with consensus of length *k* by a single state *R*_*k*_. To achieve this, state *R*_*k*_ has emission table of order *k*. According to a traditional definition [1], in a state with emission table of order *k*, the probability of a particular symbol at position *i* depends on symbols at positions *i* - *k*, …, *i* - 1. In TANTAN, however, the emission at position *i* depends only on the residue at position *i* - *k* in such a way that the two symbols *k* positions apart are equal with high probability. Thus a single state of order *k* can generate a tandem repeat with period *k*, allowing for mismatches between consecutive motif occurrences. Overall, the TANTAN submodel for a single tandem repeat of unknown length up to *K* consists of states *R*_1_, …, *R*_*K*_ connected to initial and final states, as shown in Figure 3. To allow insertions and deletions with respect to the motif consensus, TANTAN uses chains of insertion and deletion states connecting states of different order.

We have slightly modified the TANTAN model for our purposes. TANTAN uses the repeat states starting from the second occurrence of the motif, because only at that point we start to see correlations with symbols *k* positions apart. To include the first repetition of the motif in the repeat submodel, we have added a prefix path of states *P*_1_, …, *P*_*K*_ such that *P*_*k*_ is connected to *R*_*k*_ (see Figure 3).

To keep the number of parameters low, we use the same transition probability *p*_*r*_ to move from any *P*_*k*_ to *R*_*k*_ for any *k* < *K*. States *P*_*k*_ and insert states generate symbols according to the background distribution. Repeat state *R*_*k*_ assumes that the symbol at current position *i* has evolved from the symbol at position *i* - *k* according to Jukes-Cantor model with fixed time *t*. Finally, parameters *p*_*ES*_, *p*_*ER*_, and *p*_*EI*_ govern the probability of transition to end state from indel state, repeat state and init state, respectively, and *p*_*S*_, *p*_*SE*_ denote the probability of starting an indel and the probability of extending the indel.

To build a full PHMM, we use two copies of the TANTAN submodel, each emitting tandem repeat in one of the sequences. These two copies are connected to the standard three-state pair HMM representing non-repetitive sequence. We call this model TANTAN, although it differs from the model introduced by Frith [18].

Parameters of the TANTAN PHMM were set similarly as in the SFF model. Parameters *p*_*ES*_, *p*_*ER*_, *p*_*EI*_ and *t* of the TANTAN submodels were estimated by the Baum-Welch algorithm [1] on 500 repeats sampled from SFF.

The size of the SFF model is proportional to the sum of the lengths of all consensus sequences. In contrast, the size of the TANTAN model is proportional only to the length *K* of the longest represented motif. Thus SFF can be exponentially larger than TANTAN if it includes all possible consensus motifs of length up to *K*. Both SFF and TANTAN share the same overall structure, each consisting of the three-state pair HMM and a repeat submodel generating repeats separately in the two sequences. In the next section, we describe several inference methods appropriate for both of these models.

## Inference criteria and algorithms

**Figure 4:**
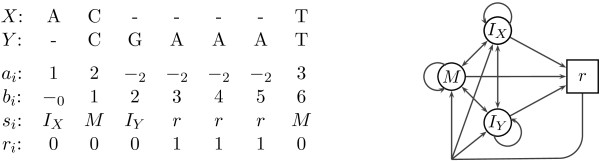
**Example of an alignment represented in our notation, together with its state and repeat annotation.** State *r* in the state sequence is a shorthand for the state *M*_1_ within submodel *R*_*j*,*Y*_ of the SFF, where *R*_*j*,*Y*_ represents motif A.

Given one of the PHMMs introduced in the previous section, and two sequences *X* = *x*_1_ … *x*_*n*_ and *Y* = *y*_1_ … *y*_*m*_, we wish to find the alignment of these two sequences best agreeing with the model. We can also annotate this alignment by labeling individual alignment columns with additional information. We start by defining an alignment and its annotation more formally (see Figure 4). *An alignment* of *X* and *Y* is a sequence of pairs (*a*_1_, *b*_1_), … (*a*_*t*_, *b*_*t*_), each pair representing one alignment column. Symbol *a*_*i*_ represents either a position in *X*, or a gap annotated with the position of the nearest non-gap symbol on the left; formally *a*_*i*_ ∈ {1, …, *n*} ∪ {-_0_, -_1_, …, -_*n*_}. To specify a valid alignment, *a*_1_ must be 1 or -_0_, *a*_*t*_ must be *n* or -_*n*_, and if *a*_*i*_ ∈ {*j*, -_*j*_}, *a*_*i*+1_ must be *j* + 1 or -_*j*_. The conditions on symbols *b*_*i*_ representing positions in sequence *Y* are analogous. The *state annotation* of an alignment is a sequence of states *s*_1_ … *s*_*t*_ such that state *s*_*i*_ generated alignment column (*a*_*i*_, *b*_*i*_). The *repeat annotation* is a binary sequence *r*_1_ … *r*_*t*_, where *r*_*i*_ = 1 if the state *s*_*i*_ generating the *i*-th column is one of the states in the repeat submodels. While the state annotation can be used with any PHMM generating the alignment, the repeat annotation is appropriate only for PHMMs explicitly modeling repeats.

We will explore several inference criteria for choosing the best alignment. To describe them, we will use the terminology of gain functions [19]; analogous notion of a loss functions is frequently used in statistics and machine learning. A *gain functionG*(*A*, *A*_*T*_) evaluates similarity between a predicted alignment *A* and the correct alignment *A*_*T*_; higher gain means that the prediction is of higher quality. Since the true alignment *A*_*T*_ is not known, we will consider the expected gain $E_{A_{T}}[G(A,A_{T})|X,Y]$ of alignment *A*, assuming that sequences *X* and *Y* were generated by our model

$$E_{A_{T}}[G(A|A_{T})|X,Y]=\underset{A_{T}}{\sum }G(A,A_{T})\mathrm{Pr}(A_{T}|X,Y).$$

In each optimization criterion, we choose a particular gain function and look for alignment *A*^∗^ maximizing the expected gain $A^{*}=\arg\underset{A}{\max}E_{A_{T}}[G(A,A_{T})|X,Y]$. Note that the gain function is only a way of defining the optimal solution; the corresponding decoding algorithm needs to be designed on a case-by-case basis.

### Decoding criteria for the three-state PHMM

For simplicity, we start with criteria for the three-state PHMM, where the state annotation is uniquely determined by the alignment itself.

#### Viterbi decoding

Perhaps the simplest gain function assigns gain +1 if the predicted alignment *A* is identical to the true alignment *A*_*T*_, and 0 otherwise. To optimize this gain function, we need to find the alignment with the highest overall probability in the model. In the simple three-state PHMM, this alignment can be found by the classical Viterbi algorithm in time *O*(*nmE*), where *E* is the number of non-zero transitions in the model.

#### Posterior decoding

While the Viterbi decoding assigns gain only if the whole alignment is correctly predicted, posterior decoding assigns gain +1 for each correctly identified alignment column. Recall that the column is a pair (*a*_*i*_, *b*_*i*_), and it is considered correct, if the same column also occurs somewhere in the true alignment. The optimal alignment under this gain function can be found by computing the posterior probability of each alignment column using the forward and backward algorithms for PHMMs, and then finding the alignment as a collection of compatible columns with the highest sum of posterior probabilities. A similar algorithm is considered for example by Lunter et al. [3], except that the column posteriors are multiplied rather than added. The running time of this algorithm is again *O*(*nmE*).

#### Marginalized posterior decoding

Lunter et al. [3] also consider a variant of posterior decoding, where column (*i*, -_*j*_) is considered correct and receives gain +1, if the true alignment contains column (*i*,-_*ℓ*_) for any value of *ℓ*. In other words, when symbol *x*_*i*_ is aligned to a gap, we do not distinguish where is the location of this gap with respect to sequence *Y*. Columns (-_*j*_, *i*) are treated symmetrically. To optimize this gain function, we again start by computing posteriors of all columns. Then we marginalize the probabilities of gap columns, effectively replacing posterior of column (*i*, -_*j*_) with the sum of posteriors of columns (*i*, -_*ℓ*_) for all *ℓ*. As before, we then find the alignment maximizing the sum of posteriors of its columns. The algorithm runs in *O*(*nmE*) time.

### Decoding criteria for the SFF Model

In more complex models, including ours, one alignment can be generated by several different state paths. Gain functions can thus take into account also the state or repeat annotation of the alignment.

#### Viterbi decoding

In more complex models, the classical Viterbi algorithm optimizes a gain function in which the alignment is annotated with the state path generating it, and gain +1 is awarded only when both the alignment and the state path are completely correct.

#### Posterior and marginalized posterior decoding

We will consider a variant of the posterior decoding, in which alignment columns are annotated by the repeat annotation, and an alignment column gets a gain +1, if the true alignment contains the same column with the same label. The only change in the algorithm is that the forward-backward algorithm produces posterior probabilities of columns annotated with the state, which are then marginalized over all states with the same repeat label. The running time is still *O*(*nmE*). Similar modification can be done for marginalized posterior decoding, where we marginalize gap columns based on both state and gap position.

Note that we might wish to completely marginalize over annotations and award gain only based on alignment. This method is however not appropriate for our models, because it would treat repeats as gaps, even if they have orthologs in the other sequence.

#### Block decoding

We will consider also a stricter gain function, which requires that repeat regions have correctly identified boundaries. We will split the alignment annotated with repeats into *blocks*, so that each maximal region of consecutive columns labeled as a repeat forms a block. Each column annotated as a non-repeat also forms a separate block. The gain function awards gain +1 for each non-gap symbol in every correctly predicted and labeled block. Correctness of non-repeat columns is defined as in the posterior decoding. A repeat block is considered correct, if exactly the same region in *X* and the same region in *Y* are also forming a repeat block in the true alignment. Note that the gain for each block is proportional to the number of non-gap symbols in the block to avoid biasing the algorithm towards predicting many short blocks.

To optimize this gain function, we first compute posterior probabilities for all blocks. Note that a block is given by a pair of intervals, one in *X* and one in *Y*. Therefore the number of blocks is *O*(*n*^2^*m*^2^). The expected gain of a block is its posterior probability multiplied by the number of its non-gap symbols. After computing expected gains of individual blocks, we can find the highest scoring combination of blocks by dynamic programming in *O*(*n*^2^*m*^2^) time.

To compute block posterior probabilities, we transform the SFF model to a generalized PHMM [20], in which all repeat states are replaced by a single generalized state *R*. In generalized HMMs, emission of a state in one step can be an arbitrary string, rather than a single character. In our case, the new state *R* generates a pair of sequences from the same distribution as defined by one pass through the repeat portion of the original SFF model. The pair of sequences generated by *R* represents one block of the resulting alignment. We call this new model the block model. Using the forward-backward algorithm for generalized HMMs, we can compute posterior probabilities of all blocks in *O*(*n*^2^*m*^2^*f*) time where *f* is the time necessary to compute emission probability for one particular block.

If we naively compute each emission separately, we get *f* = *O*(*nmE*). However, we can reduce this time for the SFF model as follows. If the SFF contains only one motif, the emission probability of sequences *x* and *y* in the *R* model is simply

$$\mathrm{Pr}\left(x,y|R\right)=\mathrm{Pr}\left(x|R_{1,X}\right)\mathrm{Pr}\left(y|R_{1,Y}\right),$$

because the model first generates *x* in the sunflower submodel *R*_1,*X*_ and then generates *y* in the model *R*_1,*Y*_. Note that these two models are connected by a transition with probability 1. In the general case, we sum the probabilities for all *k* motifs, each multiplied by the transition probability of entering that motif. To compute block emission probabilities fast, we precompute Pr(*x* ∣ *R*_*i*,*X*_) and Pr(*y* ∣ *R*_*i*,*Y*_) for all substrings *x* and *y* of sequences *X* and *Y* respectively. This can be done by the forward algorithm in *O*((*n*^2^ + *m*^2^)*E*) time. After this preprocessing, the computation of emission probability is *O*(*k*), and the overall running time of this algorithm is *O*(*kn*^2^*m*^2^ + (*n*^2^ + *m*^2^)*E*).

#### Block Viterbi decoding

The final gain function we consider is a variant of the Viterbi decoding. The Viterbi decoding assigns gain +1 for a completely correct alignment labeled with a correct state annotation. One alternative is to assign gain +1 if the alignment and its repeat annotation are completely correct. This gain function considers as equivalent all state paths that have the same position of repeat boundaries but use different motifs or different alignments of the sequence to the motif profile HMM.

In the SFF model, location of a repeat block uniquely specifies alignment within the block, because all symbols from sequence *X* must come first (aligned to gaps), followed by symbols from sequence *Y*. However, some models may emit repeat bases from the two sequences aligned to each other. We wish to abstract from exact details of repeat alignment, and consider different alignments within a repeat as equivalent. Therefore, we will reformulate the gain function in terms of blocks. The alignment labeled with repeat annotation gets a gain 1, if all its blocks are correct, where block correctness is determined as in the block decoding. This formulation is similar to the one solved by Hudek [7].

To optimize this gain function, we use the Viterbi algorithm for generalized HMMs applied to the block model, which leads to running time *O*(*kn*^2^*m*^2^ + (*n*^2^ + *m*^2^)*E*), by similar reasoning as above.

### Practical considerations

Even the fastest algorithms described above require *O*(*nmE*) time, where sequence lengths *n* and *m* can be quite high when aligning long genomic regions and the size *E* of the SFF model depends on the sum of the lengths of all repeat motifs, which can be potentially even infinite. However, we can use several heuristic approaches to make the running time reasonable.

First of all, we can use the standard technique of banding, where we restrict the alignment to some window around a guide alignment obtained by a faster algorithm. A simpler form of banding is to split the guide alignment to non-overlapping windows and realign each window separately. These techniques reduce the *O*(*nm*) factor. In practical experiments we use Muscle [21] to create guide alignments and restrict the new alignment to be within 30 base window from the guide alignment.

To restrict the size of the model, we first find tandem repeats in *X* and *Y* independently by running the TRF program [17]. Then we include in the SFF model only those motifs which appear at least once in the TRF output. If we process only relatively short windows of the banded alignment, the size of the model will be quite small. Note however, that we keep the transition probabilities entering these models the same as they are in the full SFF model. If the TRF finds a consensus not included in the original SFF model, we add its two submodels with a small probability comparable to the rarest included motifs.

These two heuristics sufficiently speed up algorithms running in *O*(*nmE*) time. The block decoding and the block Viterbi decoding need to consider all possible blocks, which is prohibitive even within short alignment windows. Therefore, we allow repeat blocks only around intervals annotated as repeats by repeat annotators. Let us assume that we have a set of intervals *T*_*X*_ annotated as repeats in sequence *X* and a set of intervals *T*_*Y*_ annotated as repeats in sequence *Y*. Note that intervals in each set are not necessarily disjoint. We restrict blocks by allowing the generalized repeat state *R* to generate the block of substrings *x* and *y* if each of these substrings is either empty or one of the intervals in *T*_*X*_ or *T*_*Y*_ respectively has both its endpoints within 10 bases from the respective endpoints of *x* or *y*. Therefore, we try at most (100|*T*_*X*_| + *n*)(100|*T*_*Y*_| + *m*) blocks.

For running algorithms with the TANTAN model, it is not necessary to know consensus motifs beforehand, but we restrict possible repeats to the same intervals as with the SFF model for block decoding and block Viterbi decoding.

**Figure 5:**
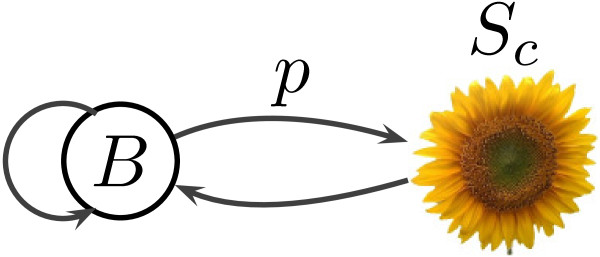
**Sunflower Repeat Finder model.** The model consists of a sunflower for a tandem repeat with a fixed consensus and a state generating non-repetitive part of the sequence.

To define repeat interval sets *T*_*X*_ and *T*_*Y*_, we first include all intervals found as tandem repeats by the TRF. However, the TRF searches for repeats in each sequence independently, which can cause problems. For example, a tandem repeat can be found in only one of the species, if it is less conserved in the other species or has there only one copy of the motif. The TRF can also find repeats in both sequences, but with slightly different consensus motifs, for example rotated by several bases. To solve these problems, we reannotate both sequences using a simple HMM which we call SRF (sunflower repeat finder). The SRF model for a motif consensus *c* consists of the sunflower submodel for motif *c* connected to a background state *B*, as shown in Figure 5. We use the Viterbi algorithm to annotate possible locations of tandem repeats with consensus motif *c* in each of the two sequences *X* and *Y* and add these locations to the sets of intervals *T*_*X*_ and *T*_*Y*_. We repeat this process for every consensus *c* discovered by the TRF in the input sequences *X* and *Y*. In this way, if the TRF discovers a tandem repeat with consensus *c* in *X*, we have a better chance to discover appropriate orthologous region matching this consensus in *Y* and add its interval to *T*_*Y*_.

The final consideration is that our PHMMs do not align tandem repeats at orthologous locations, even if they share a common evolutionary history. This might be impractical for further use. Therefore we postprocess the alignments by realigning all blocks annotated as repeats using the standard three-state PHMM. In this realignment, we also include gaps adjacent to these repeats.

## Experiments

We have compared decoding methods described in the previous section and several baseline algorithms on simulated data (see Table 1). The data set contained 200 alignments of length at least 200 each generated from the SFF model (the same model parameters were used in the sampling and for the alignments). In generating the dataset, we required that each tandem repeat had at least three copies in both species; otherwise, we would obtain many regions that would be labeled as tandem repeats, but would in fact only have a single copy. The error rate (in the first column of the table) measures the fraction of true alignment columns that were not found by a particular algorithm. It was measured only on the alignment columns that were generated from non-repeat states in the simulation, as the SFF model does not give any alignment in repeat regions.

**Table 1:** Accuracy of several decoding methods on simulated data

	**Alignment**	**Repeat**		**Block**	
**Algorithm**	**error**	**sn.**	**sp.**	**sn.**	**sp.**
SFF marginalized	**3.37%**	**95.97%**	97.78%	**43.07%**	44.87%
SFF posterior	3.53%	95.86%	97.87%	42.70%	47.37%
SFF block	3.51%	93.09%	**98.07**%	36.50%	41.67%
SFF block Viterbi	3.91%	93.26%	97.96%	35.77%	40.66%
SFF Viterbi	4.04%	95.29%	97.85%	42.70%	**48.95%**
TANTAN block	5.05%	61.38%	97.48%	0.00%	0.00%
TANTAN block Viterbi	6.17%	67.86%	96.51%	0.00%	0.00%
SFF marginalized^∗^	3.02%	98.93%	99.64%	77.01%	76.17%
SFF posterior^∗^	3.42%	98.84%	99.51%	75.91%	80.93%
SFF block^∗∗^	3.21%	97.70%	99.87%	80.66%	94.44%
SFF block Viterbi^∗∗^	3.71%	98.12%	99.85%	81.75%	92.18%
SFF Viterbi^∗^	3.94%	98.54%	99.45%	75.55%	83.47%
TANTAN block^†^	3.42%	60.45%	99.90%	0.36%	0.46%
TANTAN block Viterbi^†^	3.83%	61.74%	99.88%	0.00%	0.00%
Context	5.98%				
Muscle	5.62%				
3-state posterior	4.41%				
3-state posterior with masked repeats^††^	5.03%	99.23%	74.16%	7.66%	7.24%
3-state Viterbi (baseline)	4.78%				
SFF marginalized^◇^	3.63%	96.03%	97.74%	42.70%	43.33%
SFF marginalized^◇◇^	3.36%	95.99%	97.81%	40.88%	43.08%

^∗^: method uses the real consensus motifs.

^∗∗^: method uses the real consensus motifs and intervals from the real repeat blocks.

^†^: method uses intervals from the real repeat blocks.

^††^: Columns with at least one masked character are considered as repeats.

^◇^: Parameters for the three-state submodel were estimated from human-chicken alignment.

^◇◇^: Parameters for SFF submodel were perturbed randomly.

The methods based on the SFF model (the first block of the table) outperform the baseline method (the Viterbi algorithm on the three-state model), reducing the error rate by 15–30%. In general, the methods that score individual alignment columns are more accurate than the Viterbi-based methods, which is not surprising, because error rate as a measure of accuracy is closer to the gain function they optimize.

The SFF-based algorithms use the tandem repeat motifs predicted by the TRF, as well as approximate repeat intervals (block-based methods). The TRF predictions are not exact and may contribute to the overall error rate. We attempted to quantify this effect by using the real tandem repeat motifs and real boundary positions instead of the TRF predictions (the second block of Table 1). We can see that the use of TRF predictions indeed leaves space for improvement, with the best performing algorithm reducing the error rate by 37% compared to the baseline. It would be interesting to explore other programs for detecting tandem repeats, such as mreps [22] or ATRhunter [23].

In addition to the baseline three-state model, we have compared our approach to other alignment programs Context [15] and Muscle [21]. The Context program, which uses context-sensitive indels, was trained on a separate set of 200 alignments sampled from our model. Muscle was run with default parameters. Both programs produced alignments with higher error rate than the baseline three-state model. In case of Context, the high error rate may be due to insufficient training data or software issues.

A traditional approach to the problem of aligning sequences with repeats is to first mask repeats in each sequence independently and then perform sequence alignment. To compare to this approach, we have masked tandem repeats using TRF and then used the three-state model with posterior decoding. However, this introduced even more errors than running the same model on the unmasked sequences, ignoring the repeat issue altogether.

One could object to our tests, since we have used the same model for generating data and for testing. Therefore, we were interested in how robust is our approach to changes in model parameters. In the first experiment, we estimated parameters of the 3-state submodel of the SFF model from human-chicken alignments (human chromosome 20) instead of human-dog alignments. In the second experiment, we randomly perturbed parameters of the sunflower submodel (each parameter was changed randomly by an additive term ranging from 0.02 to 0.05). In both cases, the results have changed only slightly (Table 1), showing that our model is quite robust.

**Figure 6:**
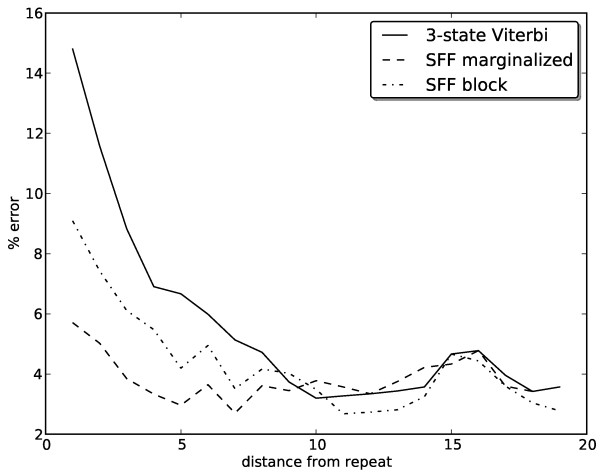
Alignment error rate of three decoding methods as a function of the distance from the nearest repeat.

The decoding methods that use the SFF model produce an alignment and a repeat annotation. Comparing annotation of each base in both sequences with the true repeat annotation sampled from the model (table columns repeat sensitivity and specificity), we note that the marginalized posterior decoding is the most sensitive, and the block decoding the most specific method. Specificity was quite high for all methods, low sensitivity for block-based methods was probably caused by wrong repeat intervals predicted by the TRF, since it improves markedly by using correct intervals.We have also compared the accuracy of predicting repeat block boundaries (table columns block sensitivity and specificity). The number of blocks with correctly predicted boundaries is quite low, most likely because usually there are many high-probability alternatives with slightly shifted boundaries. However, even though more than half of the repeat blocks have some error in the boundary placement, the SFF-based methods improve the alignment accuracy most markedly close to repeat boundaries, as shown in Figure 6. This is expected, because far from repeats, the model works similarly to the three-state PHMM.

Since the alignments were sampled from the SFF model, it is expected that the TANTAN model will be less accurate than the SFF model on this data. Somewhat surprisingly, TANTAN-based methods have even higher error rate than the baseline which uses a much simpler model. The TANTAN also had a significantly lower repeat sensitivity than the SFF and did not predict almost any block correctly. We believe that this is due to the inaccurate modeling of the first copy of the repeat, since annotations of TANTAN methods almost always skip the first copy of the repeat motif in both sequences. Merging prefix states *P*_1_, …, *P*_*K*_ in both copies of the TANTAN submodel into one chain of match states might improve the annotation of the first copy of the motif and thus possibly also the overall error rate.

To illustrate the feasibility of running our methods on real genomic data, we attempted to improve the quality of the alignment of the human chromosome 21 to the dog genome. In particular, we have downloaded mammalian alignments from the UCSC genome browser [24] and extracted the pairwise alignment of human (assembly hg19) and dog (assembly canFam2) from the multiple alignment for the human chromosome 21. In this way, we have obtained 20.8M alignment columns. We used the TRF to annotate tandem repeats in both species. Then we selected short windows covering annotated repeats and their surrounding regions (average window length was 109 bases) and realigned each window by several of the methods described above.

Since we do not know the ground truth for these alignments, only indirect evaluation is possible. We have downloaded RefSeq, ENSEMBL, VEGA and UCSC gene annotation catalogs from the UCSC genome browser and selected 2207 (possibly overlapping) transcripts that correctly matched the human reference (correct start codons, stop codons, splice sites, no in-frame stop codons). We have used our alignments to remap each transcript into the dog genome, and evaluated resulting transcripts for correctness in the dog genome using the clean_genes tool from the PHAST package [25].

**Figure 7:**
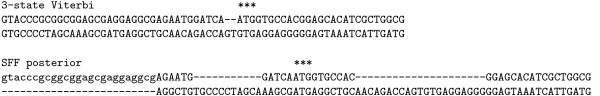
**Repeat-aware alignment may result in a significant change compared to the standard alignment methods.** Lowercase letters show a tandem repeat annotated by the SFF upstream of RefSeq gene NM_153681. While the first alignment aligned the start codon ATG in human with GTG in dog, the SFF posterior aligned it to a potential ATG start codon.

Table 2 shows success and error rates of variety of indicators of the resulting dog transcript annotation. The best performing SFF posterior approach was able to remap 750 transcripts from the human to the dog genome (compared to 730 transcripts using the baseline three-state Viterbi approach and 743 transcripts in the original UCSC alignments). The SFF-based models tended to decrease the error rate around 3’ splice sites; on the other hand, they increase the error rate in some other parameters (e.g. 5’ splice sites or frame-shifts). Many of these differences are due to small shifts in gap locations, but occasionally we see a substantial alignment change, as illustrated in Figure 7. However, problems in mapped transcripts do not always indicate an alignment error, because gene and exon boundaries may change in evolution due to indels or substitutions, particularly near 3’ ends of genes. Also note that the UCSC alignments are extracted from multiple alignments of many species, thus incorporating additional information that was not available to our alignments based on the SFF model.

**Table 2:** Evaluation of realignments of human-dog alignments (human chromosome 21)

	**Clean**	**Detected errors**					
**Algorithm**	**transcripts**	**Start**	**Stop**	**5’splice**	**3’splice**	**Nonsense**	**Frameshifts**
***Out of (dataset size)***	***2207***	***2190***	***2180***	***18898***	***18898***		
3-state Viterbi	730	292	459	**236**	401	174	**309**
UCSC alignments	743	294	427	239	415	173	348
SFF posterior	**750**	**288**	425	251	**384**	174	358
SFF marginalized	682	292	**425**	251	**384**	174	438
SFF block	714	292	432	254	388	**169**	392

## Conclusions

We have designed two new pair hidden Markov models for aligning sequences with tandem repeats and explored a variety of decoding optimization criteria for their use. The new SFF model coupled with appropriate decoding algorithm reduces the error rate on simulated data, especially around boundaries of tandem repeats. With suitable heuristics, our approach can be used to realign long genomic regions.

Our experiments are the first study comparing a variety of different decoding criteria for PHMMs. Our criteria for the SFF model optimize both the alignment and the repeat annotation. Depending on the application, one or the other may be of greater interest, and thus one may want to marginalize over all alignments and optimize the annotation, as in [8], or marginalize over labels and optimize the alignment.

Our model does not take into the account the dependencies between the repeat occurrences in the two species. A tractable model allowing such dependencies would be of great interest. Previously, we have explored the problem of aligning two sequences simultaneously to a profile HMM, but we were not able to design a simple generative model for this purpose [16].

## Competing interests

The authors declare that they have no competing interests.

## Authors’ contributions

MN and BB conceived the study and implemented the algorithms. All authors participated in algorithm design, evaluation, and in manuscript preparation. All authors read and approved the final manuscript.
